# Frühe Hilfen in Deutschland – Entwicklungsperspektiven

**DOI:** 10.1007/s00103-024-03975-6

**Published:** 2024-11-19

**Authors:** Ute Thyen

**Affiliations:** Klinik für Kinder- und Jugendmedizin, Universität zu Lübeck, Ratzeburger Allee 160, 23538 Lübeck, Deutschland

**Keywords:** Gesundheitsförderung, Sozialpolitik, Öffentliche Gesundheit, Familienförderung, Frühe Kindheit, Health promotion, Welfare policy, Public health, Family support, Early childhood development

## Abstract

Die zukünftige Entwicklung der Frühen Hilfen hängt von der strukturellen Verankerung, den Ressourcen der im Arbeitsfeld tätigen Fachkräfte und der Nutzung und Akzeptanz in der Bevölkerung ab. Zum Verständnis der Dynamik der Entwicklung werden in diesem Diskussionsbeitrag zunächst die zeitgeschichtlichen Ursprünge der Frühen Hilfen in Deutschland seit dem Beginn des 20. Jahrhunderts dargestellt. Weitere Abschnitte stellen die Wissensbestände aus verschiedenen, besonders relevanten Wissenschaften zusammen, insbesondere neurowissenschaftliche Erkenntnisse über die frühkindliche Entwicklung des Gehirns, die Bindungstheorie, Ergebnisse der Familien- und Präventionsforschung, Traumafolgenstudien sowie gesundheitsökonomische Modellierungen zum Nutzen sozialer Interventionen. Hieraus werden Anforderungen für eine Weiterentwicklung des Systems der Frühen Hilfen abgeleitet. Diese lassen sich konzeptuell als „Health-in-all-Policies“-Strategie einordnen (mit einem besonderen Schwerpunkt auf Bekämpfung der Kinderarmut), verbunden mit Interprofessionalität, inklusivem Zugang für alle Kinder und Diversitätsfreundlichkeit als Merkmale einer guten Prozessqualität. Zu einem weiteren Auf- und Ausbau der Frühen Hilfen muss der Nachweis des Nutzens durch langfristige, interdisziplinäre Forschungsprogramme in den genannten Wissensgebieten durch unabhängige Forschungsförderung sorgfältig geplant und umgesetzt werden. Aufwendige Methoden zur Evaluation von komplexen Interventionen auf individueller Ebene, bei Subgruppen und in der gesamten Bevölkerung, die auch gesundheitsökonomische Effekte sowie partizipative und qualitative empirische Forschung einbeziehen, stehen zum Teil zur Verfügung und sollten weiterentwickelt werden.

## Abstract (en)

The future development of early preventive services and intervention depends on structural anchoring, the resources of the professionals working in the field, and utilization and acceptance by the target population. In order to understand the dynamics of the development, this discussion contribution first presents the contemporary historical origins of early intervention in Germany since the beginning of the 20th century. Further sections bring together the bodies of knowledge from various relevant sciences, in particular neuroscientific findings on the early childhood development of the brain, attachment theory, results of family and prevention research, trauma studies, and health economic models on the benefits of social interventions. From this, requirements for the further growth of the early intervention system are derived. These can be conceptually classified as a “Health in all Policies” strategy (with a particular focus on combating child poverty) combined with interprofessional learning and collaboration, inclusive access for all children, and diversity-friendliness as characteristics of good process quality. To further develop and expand early interventions, proof of benefits must be carefully planned and implemented through long-term, interdisciplinary research programs in the aforementioned fields of knowledge through independent research funding. The complex methods for evaluating complex interventions at the individual level, subgroups, and the entire population, including health economic effects and participatory and qualitative empirical research, are partly available and should be further developed.

## Hintergrund

**Abb. 1:**
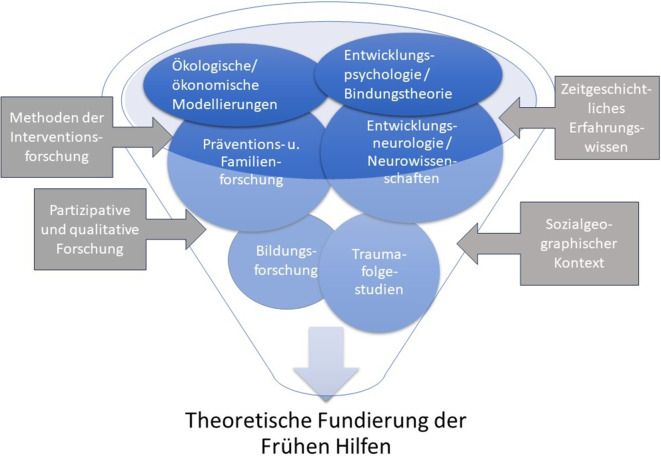
Theoretische Fundierung der Frühen Hilfen. (*Quelle*: eigene Abbildung)

Programme und Angebote für Frühe Hilfen basieren auf sozialpsychologischen Theorien und empirischem Wissen einerseits und gesundheits- und sozialpolitischen Zielsetzungen andererseits. Diese sollen in diesem Diskussionspapier vor dem Hintergrund des historischen Kontextes skizziert werden. Für die Diskussion von Entwicklungsperspektiven und -möglichkeiten der Frühen Hilfen sind das Verständnis der spezifischen Entwicklungslinien in Deutschland und das aktuell verfügbare Wissen hilfreich. Dazu folgt ein kurzer historischer Rückblick, sodann eine Darstellung des aktuellen Forschungsstandes in den Feldern Neurowissenschaften, Entwicklungspsychologie, Familien- und Präventionsforschung sowie gesundheitsökonomische Modellierungen zum Nutzen sozialer Interventionen (Abb. 1).

## Historischer Rückblick: Soziale Fürsorge für Säuglinge und Kleinkinder

Der Gedanke der Förderung und Anerkennung des Kindes als Person stammt aus den Anfängen des 19. Jahrhunderts. Während der frühen Industrialisierung entstand das moderne Proletariat der Fabrikarbeiter:innen, Armut und Verelendung führten zur Diskussion der „sozialen Frage“, d. h. der Frage nach der Verteilung des Reichtums und nach Chancengerechtigkeit für alle Bürger:innen. In der zweiten Hälfte des 19. Jahrhunderts entwickelte sich eine moderne Sozialpolitik mit Sozialversicherungssystemen, Gründung von Wohlfahrtsverbänden und sozialen Vereinen. Diese sozial- und gesundheitspolitisch motivierten Initiativen des späten 19. und beginnenden 20. Jahrhunderts sind bevölkerungsmedizinisch motiviert und standen einerseits in einem moralischen, andererseits einem volkswirtschaftlichen Begründungszusammenhang [1].

In den Jahren der Weimarer Republik war ein großer Aufwuchs von qualifizierten Ausbildungsgängen zu Säuglingspflegerinnen und Kinderkrankenpflegerinnen unter der Initiative von Kinderärzt:innen zu beobachten, die auch die frühen Grundsteine für die Sozialpädiatrie legten [2]. Das folgende Zitat des jüdischen Kinderarztes Arthur Schlossmann, der das erste Säuglingskrankenhaus in Dresden leitete, drückt eine Zielsetzung aus, die auch den heutigen Frühen Hilfen zu eigen ist:

„Säugling zu sein, ist in unserer Zeit eine Lust. Was auf dem Verordnungswege möglich ist, geschieht, um dem jungen Weltbürger sein Leben lebenswert zu machen und um ihm alle Steine des Anstoßes aus der Wiege zu räumen“ [3].

Der bevölkerungsmedizinische Ansatz (ver)führte auch dazu, im Sinne der damals modernen Ideen der Eugenik verstanden zu werden: „Vollwertiges, gesundes Leben“ sollte gefördert werden, nicht aber das von Kindern mit angeborenen Behinderungen, die „Taubstummen, Spastiker und Idioten“. Von dieser Position war es für nicht wenige Kinderärzt:innen nur ein kleiner Schritt, an der Vernichtung „unwerten Lebens“ mitzuwirken [4].

Parallel zu ärztlich-pflegerischen Bemühungen um eine Senkung der Säuglings- und Kleinkindersterblichkeit und der Gesundheitsförderung der kleinen Kinder entstanden die großen bis in die heutige Zeit wirkenden reformpädagogischen Konzepte für Kinder mit und ohne Behinderungen, stellvertretend seien hier nur Maria Montessori, Emmi Pickler oder Janusz Korczak zu nennen [2]. Die bis heute vorbildlichen und wegweisenden Konzepte der Frühen Hilfen waren also historisch gesehen von Anfang an ein Tätigkeitsfeld, das mindestens durch Medizin, Pflege, Pädagogik und soziale Arbeit getragen und gespeist wurde.

**Abb. 2:**
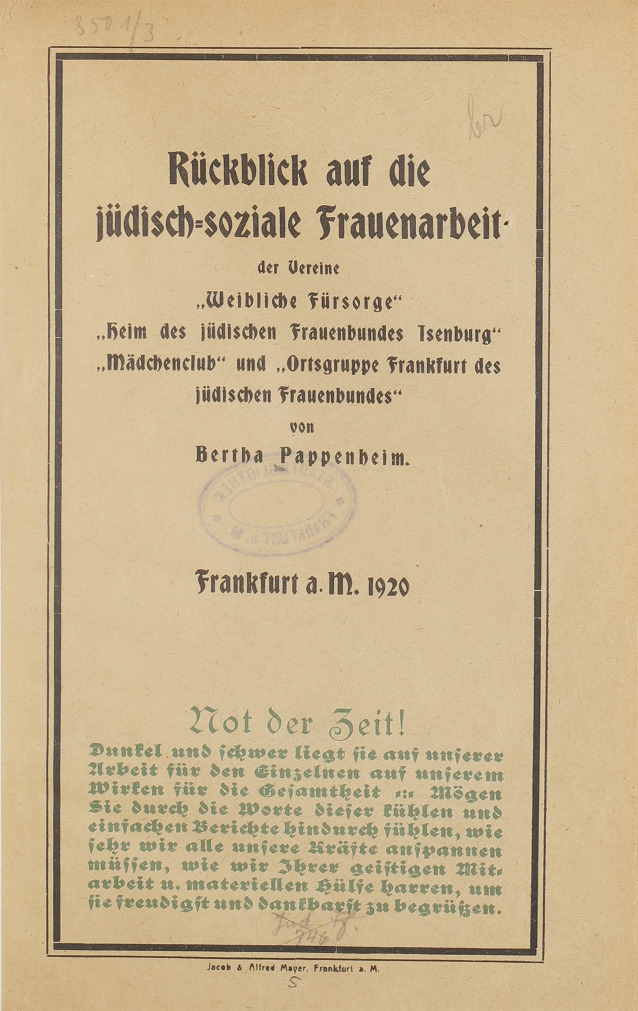
Soziale Arbeit in der Weimarer Republik. Deckblatt des Berichts von Bertha Pappenheim: Rückblick auf die jüdisch-soziale Frauenarbeit der Vereine „Weibliche Fürsorge“, „Heim des jüdischen Frauenbundes Isenburg“, „Mädchenclub“ und „Ortsgruppe Frankfurt des jüdischen Frauenbundes“, Frankfurt a. M. 1920 [4]. Digitalisiert durch die Universitätsbibliothek J.C. Senckenberg, Frankfurt am Main, 2011 (https://sammlungen.ub.uni-frankfurt.de/urn/urn:nbn:de:hebis:30:1-161635)

Auch ehrenamtliches Engagement spielte schon früh eine große Rolle, insbesondere organisiert durch Wohlfahrtsverbände und Initiativen wie die jüdische Frauenarbeit. Der Hilferuf „Not der Zeit!“ von Bertha Pappenheim [5] enthält wesentliche Aspekte der notwendigen Ausstattung und der Zielsetzung der damaligen Fürsorgebemühungen inmitten einer bedrückenden sozialen und wirtschaftlichen Situation (Abb. 2).

Während der Zeit des Nationalsozialismus wurde die Säuglingsfürsorge weiter ausgebaut, nun aber mit einer vollständigen Ausrichtung auf eugenische Ziele und Vermehrung des Kinderreichtums in „arischen“ Familien [4]. Die weiteren Entwicklungen des öffentlichen Gesundheitswesens nach dem Ende des Krieges verliefen in der Bundesrepublik Deutschland und der Deutschen Demokratischen Republik (DDR) sehr unterschiedlich. In den Nachkriegsjahren wurde in der DDR ein umfassendes System der staatlichen Fürsorge und Versorgung gemäß den sozialistischen Zielen aufgebaut. Bereits in den ersten Lebensjahren war eine außerfamiliäre Betreuung in Kinderkrippen üblich, damit Mütter wie Väter am Arbeitsleben teilnehmen konnten. Alle Fürsorgemaßnahmen für werdende Mütter, Unterstützung von Familien und Gesundheitsförderung waren hier staatlich organisiert [6].

In Westdeutschland wurden noch vorhandene staatliche Fürsorgeprogramme des öffentlichen Gesundheitswesens (z. B. Mütterberatung in den Gesundheitsämtern) in den 1970er- bis 1980er-Jahren stark abgebaut und viele Aufgaben in den Zuständigkeitsbereich der gesetzlichen und privaten Krankenkassen, d. h. in die hausärztliche Versorgung, verschoben [7].

Nach der Wiedervereinigung 1990 wurden die sozialpolitischen Konzepte aus der ehemaligen BRD auf die Länder der ehemaligen DDR ausgedehnt verbunden mit einem Abbau der vorhandenen staatlichen Systeme. Im Jahr 2000 erfolgt eine Änderung des Bürgerlichen Gesetzbuches mit dem Recht des Kindes auf eine gewaltfreie Erziehung.

Im Jahr 2006 erfuhr der Tod eines 2‑jährigen Jungen in Bremen durch Kindesmisshandlung sehr starke mediale Aufmerksamkeit, weitere Fälle folgten. Die damit verbundenen politischen Debatten haben zur Einführung „verbindlicher“ Früherkennungsuntersuchungen durch Kinder- und Jugendärzt:innen geführt [8]. 2007 wurde das Nationale Zentrum Frühe Hilfen (NZFH) an der Bundeszentrale für gesundheitliche Aufklärung (BZgA) in Kooperation mit dem Deutschen Jugendinstitut (DJI) durch das Bundesministerium für Familie, Senioren, Frauen und Jugend (BMFSFJ) eingerichtet und der Ausbau von Netzwerken der Frühen Hilfen auf kommunaler Ebene gefördert [9]. Beide Initiativen wurden zunächst als „Frühwarnsysteme“ zur Erkennung drohender Kindesmisshandlung und -vernachlässigung konzipiert. Die Fachöffentlichkeit und der begleitende wissenschaftliche Beirat sahen die Engführung der Frühen Hilfen auf den Kinderschutz eher kritisch und maßen der Ressourcenstärkung in den Familien eine ebenso große Bedeutung wie der Risikoerkennung bei [10, 11].

Das System der „Frühförderung“ als Leistung der Eingliederungshilfe für Kinder mit (drohenden) Behinderungen entstand in den 1970er-Jahren und bestand gleichermaßen in der DDR und der BRD, ihr Fortbestand wurde durch die zusätzlichen Angebote der Frühen Hilfen nicht eingeschränkt. Trotz zunehmender Kooperationsbemühungen und -erfolge insbesondere auf kommunaler Ebene handelt es sich um Angebote mit verschiedenen Zugangswegen, Finanzierungslogiken und unterschiedlichen Regelungen in den Sozialgesetzbüchern (SGB, Frühe Hilfen SGB VIII und Frühförderung SGB IX) für teilweise überlappende Zielgruppen. Insofern treffen viele der Themen dieses Beitrages sowohl auf die Frühen Hilfen als auch auf die Frühförderung zu, fokussiert werden jedoch die Frühen Hilfen. Im Sinne der inklusiven Kinder- und Jugendhilfe ist in den nächsten Jahren eine zunehmende Integration der Systeme zu erwarten [12].

## Kinderrechtliche Orientierung

Frühe Hilfen setzen ein Grundverständnis von Kindern als Grundrechtsträger:innen voraus. Die Weiterentwicklung und Ausdehnung der Idee der universellen Menschenrechte fällt in die erste Hälfte des 20. Jahrhunderts. 1924 veröffentlichte der Völkerbund die sogenannte Genfer Erklärung, die „Children’s Charta“, in der grundlegende Rechte für Kinder formuliert wurden. Diese wurde nach mehrjährigen Vorarbeiten durch die Generalversammlung der Vereinten Nationen 1959 in eine Erklärung der Rechte des Kindes überführt. 1989 legte die UN-Generalversammlung das bis heute wichtigste Menschenrechtsdokument für Kinder, die Konvention über die Rechte des Kindes, vor, die 1992 von Deutschland unterzeichnet wurde. Die Aufnahme der spezifischen Kinderrechte nach der UN-Konvention in die Verfassung der Bundesrepublik Deutschland, dem Grundgesetz, ist allerdings noch immer nicht erfolgt [13].

## Entwicklungsneurologie: neurobiologische Forschungsergebnisse

Der Slogan: „die ersten 1000 Tage zählen“, bezieht sich auf die rasante Entwicklung des bei der Geburt sehr unreifen Gehirns eines Kindes. Die frühe neuronale Vernetzung im kindlichen Gehirn bildet die Grundlage für die psychische wie auch die kognitive Entwicklung. Die moderne Neurobiologie hat insbesondere mit bildgebenden Verfahren und Untersuchungen der Regulation von Stresshormonen empirische Belege für die nachhaltigen negativen Auswirkungen von sogenannten toxischen Stressoren erbracht. Damit sind Einflüsse gemeint, die die Kompensationsmöglichkeiten des heranreifenden Organs überschreiten und die typische physiologische Entwicklung beeinträchtigen. Von großer Bedeutung sind hierbei Substanzen, die schädigend auf das ungeborene Kind einwirken – Alkoholkonsum während der Schwangerschaft ist die wichtigste Ursache für angeborene Entwicklungsstörungen bei Kindern in Deutschland [14]. Aber auch schwerer pränataler psychosozialer Stress bei Schwangeren kann zur langfristigen Störung der Stressregulation des Kindes führen [15, 16]. Shonkoff und Kollegen haben eine umfassende Theorie zum Zusammenwirken von genetischen Faktoren, früher Vernachlässigungs- oder Gewalterfahrung und zur Bedeutung der Zeitpunkte von Belastungen in Bezug auf das Entwicklungsalter und den sozialen Kontext vorgelegt [17]. Sie gehen davon aus, dass alle Maßnahmen, die „responsive caregiving“ fördern und die zeitsensitiv und kontextbezogen eingesetzt werden, maximalen Benefit für die Entwicklung und Gesundheit von Kindern haben werden und in der Lage sind, Risiken auszugleichen. „Responsive caregiving“ meint die Fähigkeit einer Bezugsperson, die vorsprachlichen Signale eines Kindes feinfühlig wahrzunehmen sowie angemessen und verlässlich darauf zu reagieren. Die Erkenntnisse haben sich auch das Kinderhilfswerk der Vereinten Nationen (UNICEF), die Weltbank und die Weltgesundheitsorganisation (WHO) zu eigen gemacht mit einem Rahmenprogramm zur Förderung in der Frühen Kindheit [18].

## Entwicklungspsychologie: Bindungstheorie

Die empirische entwicklungspsychologische Forschung kam sowohl in Bezug auf die sprachlich-kommunikative wie auch die sozialemotionale Entwicklung zu der Erkenntnis, dass es frühe Entwicklungsfenster gibt, in denen Lernprozesse leichter möglich sind. Dabei gehen andere Entwicklungsschritte voraus und es werden weiter folgende begünstigt. John Bowlby begründete Ende der 1960er-Jahre die Theorie über die Bindung von Säuglingen und Kleinkindern an ihre Bezugspersonen, sie wurde durch Mary Ainsworth mit empirischen Forschungsergebnissen gestützt [19]. Beziehungserfahrungen und die dadurch innerpsychisch entstehenden Bindungsmuster haben eine große Bedeutung für die Muster der Selbststeuerung und der sozialen Interaktionen. Die Bindungstheorie und insbesondere die 4 postulierten Bindungsmuster werden zum Teil wegen ihrer Zentrierung auf westliche Kulturen kritisiert [20, 21]. Dennoch bietet die Bindungstheorie eine wesentliche Grundlage für Fachkräfte aus der Pädagogik und den Frühen Hilfen. Die zunehmende frühe Berufstätigkeit von beiden Elternteilen erfordert die Berücksichtigung der Bindungstheorie auch in Krippen und Kindertagespflege.

Der Aufbau sicherer oder zumindest stabiler Bindungsmuster und damit eines Vertrauens in soziale Beziehungen bildet die Grundlage für die Befähigung zur Empathie, zum prosozialen Verhalten und zur Bewältigung von Krisen und Herausforderungen. Die Bindungsprozesse können sowohl durch Erkrankungen, Temperament oder Regulationsstörungen des Säuglings als auch durch psychische Belastungen und Erkrankungen der Eltern beeinträchtigt werden (siehe dazu den Beitrag von Zietlow und Krumpholtz in diesem Themenheft). Fachkräfte der Frühen Hilfen achten daher sowohl auf Signale des Säuglings, die auf nicht gelingende Interaktionen hinweisen, als auch auf die Kompetenzen und das Wohlbefinden der Bezugspersonen [11, 12].

## Präventionsforschung in und mit Familien

Die Förderung und gelingende Erziehung der Kinder hängen in starkem Maße von den Ressourcen ihrer Bezugspersonen ab [22, 23]. Dabei handelt es sich um persönliche Ressourcen, aber auch Ressourcen im Umfeld der Familie. Kinder, die in Familien mit Instabilität, Verlusten von Bindungspersonen und in Armutsverhältnissen aufwachsen, zeigen einen um etwa 10 Punkte niedrigeren Intelligenzquotienten und verschiedene psychische Auffälligkeiten im Vergleich zu Kindern ohne solche Risiken [24].

Verschiedene Programme zur frühzeitigen Unterstützung und Beratung von Eltern im Rahmen von Hausbesuchen konnten zeigen, dass sich insbesondere in Familien mit psychosozialen Belastungen und geringem Bildungsgrad signifikante Verbesserungen im Hinblick auf die Entwicklung der Kinder als auch das psychische Wohlbefinden von Müttern erreichen lassen, allerding mit einer hohen Heterogenität zwischen verschiedenen Programmen und unterschiedlichen Settings [25]. Sehr deutliche und vor allem nachhaltige Effekte zeigte insbesondere das von David Olds in den 1990er-Jahren initiierte Hausbesuchsprogramm „The Nurse-Family Partnership“, das Familien von der Schwangerschaft bis zum zweiten Geburtstag des Kindes angeboten wurde. Die Nachbeobachtung bis zum Alter von 9 Jahren ergab eine Verringerung der Folgegeburten bei den Müttern, eine Vergrößerung der Abstände zwischen der Geburt des ersten und zweiten Kindes, eine Erhöhung der Stabilität der partnerschaftlichen Beziehungen und eine Verbesserung der Schulleistungen der Kinder in der Grundschule. Noch 18 Jahre später zeigten sich signifikante Unterschiede im Bildungserfolg der Kinder, eine gesundheitsökonomische Effektivität des Programms sowie geringere Raten von Kindesmisshandlung [26]. Zunehmend hat sich bei der Erprobung von Angeboten und Programmen gezeigt, dass es sich bei der Gesundheitsförderung von Kindern um komplexe Interventionen handelt, die immer die Einbindung und Stärkung der gesamten Familien voraussetzen [27, 28].

Familienförderung und Empowerment erfolgen am besten eingebettet in die Lebenswelt und das Quartier. Insbesondere Gestaltung des öffentlichen Raumes, einschließlich der Kindertagesstätten und Schulen sowie Freizeitanlagen und Verkehrsgestaltung, Zugang zu gesunder Ernährung und Wasser, Umweltgerechtigkeit und Klimaanpassung sind Maßnahmen der Verhältnisprävention, ohne die eine auf Verhaltensänderungen ausgerichtete Prävention nicht sinnvoll ist [29, 30].

Eine wohnortnahe und qualitätsgesicherte außerfamiliäre Betreuung in Kinderkrippen mit entsprechenden frühkindlichen Bildungsangeboten kann dazu beitragen, die Entwicklung des Kindes zu fördern [31], Eltern psychosozial zu entlasten, Erwerbsarbeit insbesondere bei alleinerziehenden Eltern zu ermöglichen und damit auch Kinderarmut in diesen Familien zu bekämpfen [32]. Internationale Studien stützen diese Ergebnisse insbesondere mit Langzeitstudien [33].

## Volkswirtschaftliche Modellierungen

Die wohl bekannteste Modellierung des Nutzens von Investitionen in die frühe Kindheit stammt von dem Wirtschaftswissenschaftler James Heckman, der in Chicago das „Center for the Economics of Human Development“ leitet und im Jahr 2000 den Wirtschaftsnobelpreis für seine Methoden zur Untersuchung von spezifischen Subgruppen der Gesellschaft als Grundlage für eine evidenzgeleitete Bildungspolitik erhielt. Er konnte zeigen, dass Investitionen in die ersten Lebensjahre den höchsten „return of interest“, d. h. die höchste Effizienz bei der Verteilung von Fördermitteln, haben [34].

Eine deutsche Studie, die die Kosten nicht erkannter oder nicht angemessen behandelter früher Traumatisierung aufgrund von Misshandlung und Vernachlässigung berechnet, zeigt, dass durch Prävention erhebliches Leid, aber auch großer volkswirtschaftlicher Schaden abgewendet werden könnte [35]. Auch im Bereich der frühkindlichen Bildung sind Modellierungen erfolgt, die den volkswirtschaftlichen Nutzen eines frühen Bildungsangebotes, z. B. durch Besuch einer Kinderkrippe ab dem 2. Lebensjahr, nachweisen [36], entsprechende Modellierungen für die Wirkungen der Frühen Hilfen stehen noch aus.

## Aktuelle Strukturen

Deutschland verfügt über ein reiches und breites Unterstützungs- und Versorgungssystem für Familien. Allerdings sind die Strukturen gekennzeichnet durch ein hohes Maß an regionalen Unterschieden durch die Zuständigkeit der Länder, durch die kommunale Ausgestaltung der Regelungen und Gesetze sowie die heterogene Handhabung durch die Träger der Sozialversicherung. In den Diskussionen um die Kindergrundsicherung wurde offenbar, dass zahlreiche finanzielle Förderprogramme aufgrund mangelnder Transparenz, Zugangsbarrieren durch Diskriminierung und bürokratischen Aufwands von den Familien nicht in Anspruch genommen werden. Dies kennzeichnet nicht nur ein Dilemma für betroffene Familien, auch Fachkräfte der Frühen Hilfen sind oft überfragt, wenn es um die Aspekte soziale Sicherung, sichere Unterkunft, Bildungschancen der Kinder oder Eingliederungsmaßnahmen geht. Diese kleinteilige, oft kurzfristige und von aktuellen Krisen immer wieder gestörte Familienförderung stellt eine erhebliche Barriere für die Effektivität der Hilfsangebote der Frühen Hilfen dar [37].

**Abb. 3:**
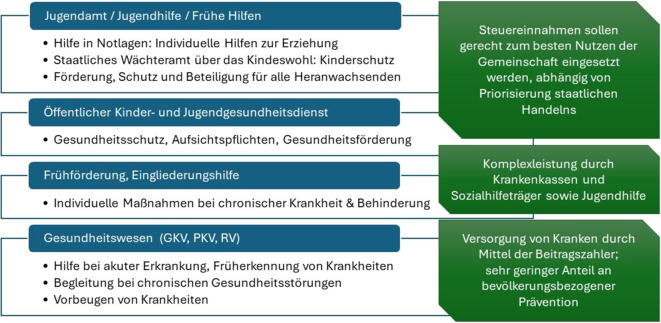
Systemlogiken der staatlichen Daseinsfürsorge und der Sozialversicherungssysteme. (*Quelle*: eigene Abbildung). *GKV* Gesetzliche Krankenversicherung, *PKV* Private Krankenversicherung, *RV* Rentenversicherung

Innerhalb der interprofessionellen Zusammenarbeit kann eine vertiefte Kenntnis der Systemlogiken von steuerfinanzierten staatlichen Hilfen und den Unterstützungsmöglichkeiten aus den Sozialversicherungssystemen zu einem besseren Verständnis der Handlungslogiken von Fachkräften aus anderen Bereichen und der Zusammenarbeit führen (Abb. 3). Das Gelingen von Hilfeprozessen ist voraussetzungsreich: Ethische Aspekte und sozialstaatliche Verpflichtungen, Spannungsfelder zwischen Hilfe und Kontrolle, mögliche Stigmatisierungsprozesse durch Hilfeannahme, ernstgemeinte Partizipation der Zielgruppen und Barrieren in Zugangswegen müssen bedacht werden.

## Entwicklungsperspektiven

**Abb. 4:**
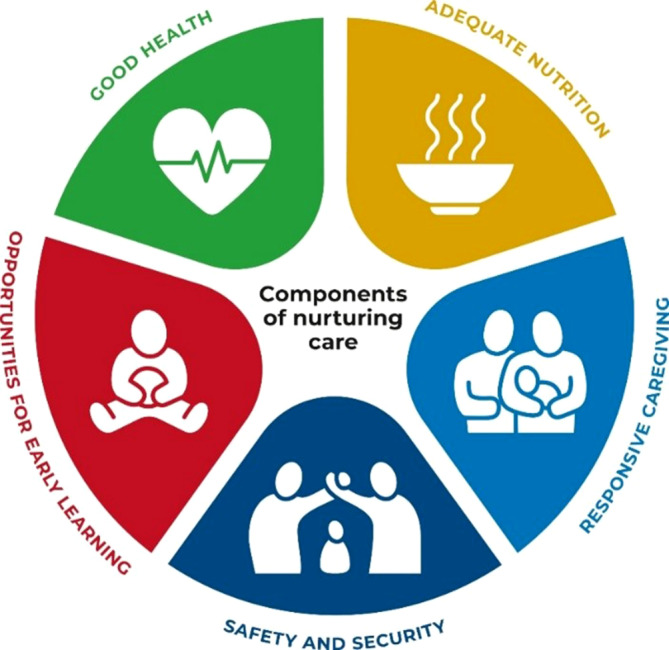
Die 5 Komponenten einer fürsorglichen Versorgung zur Förderung der frühkindlichen Entwicklung (Nurturing Care Framework der Vereinten Nationen): guter Gesundheitszustand, adäquate Ernährung, bedarfsgerechte Pflege, Sicherheit und Schutz, Möglichkeiten für frühes Lernen. (*Quelle*: https://nurturing-care.org/, Abbildung frei verfügbar über https://nurturing-care.org/wp-content/uploads/2020/12/NCF_new-logo-with-labels-1000.jpg, Zugegriffen am 15.10.2024)

*Frühe Hilfen als Teil einer nationalen und internationalen Public-Health-Strategie.* Grundsätzlich sind die Frühen Hilfen ein Teil der Public-Health-Strategie des Bundes, der Länder und der Kommunen [38]. Sie wirken in den zentralen Handlungsfeldern der Prävention und Gesundheitsförderung und wirken auch in die Bereiche Infektions- und Katastrophenschutz hinein durch Vorhaltung einer kritischen Infrastruktur für familiäre Unterstützung. Die Rahmung einer Public-Health-Strategie für Kinder, ihre Mütter und Väter erfolgt durch die Ziele für nachhaltige Entwicklung (Sustainable Development Goals) der Vereinten Nationen [39]. Zentrale Bestandteile sind Armuts- und Diskriminierungsbekämpfung, Bildungs- und Chancengerechtigkeit, gesunde Ernährung und Klimaanpassung und in Bezug auf kleine Kinder insbesondere ein förderndes familiäres und gesellschaftliches Umfeld (Nurturing Care Framework; Abb. 4).

Eine Anerkennung der vorrangigen Berücksichtigung des Kindeswohls bei allen politischen Entscheidungen sollte als eigenständiger Grundrechtsartikel verankert werden [13]. Dies ist auch eine zentrale Lehre aus der Bewältigung der COVID-19-Pandemie, in der Kinder systematisch benachteiligt wurden [40].

Hier sollen nun die 6 wichtigsten Eckpunkte benannt werden, die eine strukturelle und konzeptuelle Weiterentwicklung der Frühen Hilfen in Deutschland ermöglichen und stärken.

### Health in all Policies

Frühe Hilfen sind als Bestandteil einer ganzheitlichen Familienpolitik in Deutschland zu sehen. Eine solche Strategie folgt einem Ansatz der „Gesundheit in allen Politikbereichen“ (Health in all Policies), weil die Gesundheitsförderung von Kindern in ihren Lebenswelten mehr verlangt als nur eine Gesundheitsversorgung durch das medizinische System und eine Gesundheitsförderung durch die Jugendhilfe [38]. Städteplanung, Armutsbekämpfung, Anpassung an den Klimawandel, Vorhaltung von Betreuungseinrichtungen, Angebote für frühe Bildung und Förderung, Inklusion von Kindern mit besonderen gesundheitlichen Herausforderungen, Demokratieförderung und Abbau von Diskriminierung bilden den Rahmen für eine wirksame *Verhältnisprävention,* die eine individuelle Arbeit mit den Familien und Bezugspersonen im Sinne einer Verhaltensänderung und Stärkung der Selbstwirksamkeit flankiert und unterstützt [30]. In diesem Sinne sollen die Frühen Hilfen ein zentraler Bestandteil der *Neustrukturierung der Public Health Governance* durch die Bundesregierung, die für den öffentlichen Gesundheitsdienst zuständigen Länder und die kommunale Selbstverwaltung sein. Voraussetzung für jede konzeptuelle Weiterentwicklung ist eine kontinuierliche, verlässliche und auskömmliche Finanzierung durch den Bund über die Bundesstiftung Frühe Hilfen, die Länder und die Beiträge der Kommunen.

### Aus‑, Fort- und Weiterbildung

Auf der Ebene der Aus‑, Fort- und Weiterbildung der im Feld der Frühen Hilfen tätigen Fachkräfte muss sichergestellt werden, dass ausreichend viele Menschen in den entsprechenden Ausbildungsberufen und Studiengängen ausgebildet werden (soziale Arbeit, Pflege, Gesundheits- und Hebammenwissenschaften u. a. therapeutische und pädagogische Berufe). Zudem sollte im Feld der Frühen Hilfen die Befähigung zur interdisziplinären Zusammenarbeit gegeben sein, diese kann am besten durch gemeinsame Aus‑, Weiter- und Fortbildungsmodule in den Ausbildungs- und Studiengängen vermittelt werden. Inhalte solcher für alle Fachkräfte gleichermaßen wichtigen Module wären u. a.: systemischer Blick auf Familien, Lebensspannenansatz, Diversitätssensibilität, Teilhabeorientierung, Ebenen der Prävention, Health-in-all-Policies-Ansatz und Inklusionsfreundlichkeit [41].

### Inklusion

Die Inklusion von Kindern mit und ohne körperliche, geistige und seelische Funktionseinschränkungen darf nicht entlang von festgestellten Behinderungen und sozialgesetzlichen Zuständigkeiten oder vorhandenen Institutionen verhandelt werden. Eine genaue Untersuchung der Entwicklungsmöglichkeiten und des zusätzlichen Unterstützungsbedarfs muss gemeinsam mit dem betroffenen Kind und den Bezugspersonen entwickelt und priorisiert werden. Alle in den verschiedenen Feldern tätigen Fachkräfte und die dazugehörigen Institutionen bilden eine Gemeinschaft, die die Bedürfnisse des Kindes und der Familie bestmöglich befriedigt [11, 12, 37]. Dazu kann es erforderlich sein, sektoren- und sozialgesetzbuchübergreifend tätig zu werden, verschiedene Finanzierungsmöglichkeiten zu nutzen und flexibel Pläne und Angebote im Entwicklungsverlauf anzupassen. Nur so ist eine Inklusion dieser Kinder im Sinne des Übereinkommens der Vereinten Nationen über die Rechte von Menschen mit Behinderungen (UN-Behindertenrechtskonvention, UN-BRK), die 2009 von Deutschland ratifiziert wurde, möglich.

### Versorgungsbedarfe bei Flucht und Migration

In einem Einwanderungsland wie Deutschland sollten die Versorgungsbedarfe von nichtdeutschen, geflüchteten oder migrierenden Kindern besonders in den Blick genommen werden [42]. Ihre gesundheitlichen und Bildungschancen hängen sehr stark davon ab, wie rasch eine Integration in Bildungs- und Betreuungsangebote erfolgen kann; sekundäre Traumatisierung durch Aufenthalte in Aufnahmelagern oder gesellschaftliche Diskriminierung müssen vermieden werden. Die Integration wird insbesondere dadurch erreicht, dass die Eltern rasch eine Arbeit aufnehmen und eine sprachliche Befähigung erlangen.

### Armutsbekämpfung

Das Aufwachsen in Armut stellt den bedeutsamsten und einflussreichsten Faktor für ungleiche Chancen auf eine gesunde Entwicklung dar. Da es sich um einen potenziell veränderbaren Faktor handelt, müssen größere gesellschaftliche Anstrengungen unternommen werden, um diesen Risikofaktor abzuwenden [43]. Die Diskussion sollte nicht auf Armutsbekämpfung allein ausgerichtet sein, sondern auch die Verteilung und ggf. Begrenzung von Reichtum in den Blick nehmen [44].

### Forschungsförderung

Bei Programmen und Angeboten, die gesellschaftliche Gruppen erreichen sollen, die sich in einer besonders belasteten Lebensphase befinden, muss der Nutzen der gesellschaftlichen Interventionen wissenschaftlich nachgewiesen werden. Dies kann nur durch eine sehr breit angelegte *Forschungsförderungsstrategie* gelingen, die mindestens die in diesem Beitrag skizzierten Wissenschaftsbereiche einbezieht und für die einzelnen Forschungsfragen angemessene Förderlaufzeiten ermöglicht, z. B. 10 Jahre. Vertiefende Einzelstudien in spezifischen Forschungsbereichen sind zur Beantwortung offener Fragen ebenso erforderlich wie große, kollaborative Initiativen zur Evaluation des Gesamtnutzens für die Bevölkerung. Daher ist auch eine an Ressorts gebundene Forschungsförderung hier nicht sinnvoll, sollten doch multisystemische Entwicklungen über die Zeit und über verschiedene Bevölkerungsgruppen untersucht werden. Angesichts des derzeit bereits bestehenden Wissens scheinen begrenzte Projekte und ihre Evaluationen keinen weiteren wissenschaftlichen Mehrwert zu erbringen. Die erforderlichen Methoden zur Erforschung komplexer Interventionen wurden etabliert, bedürfen aber einer kontinuierlichen Weiterentwicklung. Auch hier bietet sich eine Orientierung an internationalen Studien zur Implementierung und Transformation von Systemen an [27, 28]. Die Qualität der Interventionen wird in hohem Maße von kontextuellen Faktoren wie Infrastruktur, sozialen Normen, Nachfrage und Interesse der Zielgruppen beeinflusst, sodass die Zielgruppen als Expert:innen ihrer eigenen Lebenswelten bereits in die Entwicklung der Fragestellungen, des Designs, der Durchführung und der Interpretation der Ergebnisse bestmöglich mit eingebunden werden sollen.

## Fazit und Empfehlungen

Die Frühen Hilfen in Deutschland wurden vergleichsweise spät, erst in den 90er-Jahren des 20. Jahrhunderts in einer flächendeckenden Angebotsstruktur in Deutschland etabliert. Inhaltlich sind die Frühen Hilfen im Bereich der Ressourcenstärkung und Gesundheitsförderung angesiedelt. Da es sich um jeweils mit den Familien partizipativ zu klärende passgerechte Hilfen handeln muss, gehört ein sehr breites Spektrum der Angebote, Hilfen, Netzwerke und Institutionen zu den Frühen Hilfen. Die Ausgestaltung bleibt der kommunalen Vielfalt überlassen, daher gibt es in Deutschland im Unterschied zu manch anderen Ländern keine einheitliche Programmstruktur und kein „Markenzeichen“ für die Frühen Hilfen. Dadurch ist aber auch ein sehr flexibles Arbeitsfeld entstanden, nah an den Familien und ihren Bedarfen und ständig bereit zur Reflexion und Weiterentwicklung. Die Fachkräfte der Frühen Hilfen haben über das Nationale Zentrum Frühe Hilfen leichten Zugang zu allen oben skizzierten Wissensbeständen, das NZFH bereitet diese auf einer Lernplattform^1^ zeitnah und für die Praxis nutzbringend auf. „Markenkerne“ sind bei aller Vielfalt der feinfühlige Umgang mit Kindern, Müttern und Vätern sowie die Bereitschaft zur Zusammenarbeit mit anderen Fachkräften. Aus dieser Sicht ergeben sich fachlich keine Barrieren für die weitere Entwicklung der Frühen Hilfen in Deutschland. Wesentliche Barrieren oder Gefahren bestehen in einer nicht nachhaltigen, d. h. nicht verlässlichen strukturellen und finanziellen Absicherung und einer fehlenden Governance im Sinne eines umfassenden ressort- und sozialgesetzbuchübergreifendes Regierungshandelns. Eine unabhängige breite und langfristige Forschungsförderung ist erforderlich, um wissenschaftliche Evidenz für den Nutzen der Angebote zu gewinnen. Dadurch werden einerseits Anpassungen und reflexives Lernen innerhalb des Systems ermöglicht und andererseits Strukturen und Qualitätsstandards gesichert.

## Footnotes

1 https://www.fruehehilfen.de/qualitaetsentwicklung-fruehe-hilfen/qualifizierung/lernplattform-fruehe-hilfen/; Zugriff: 15.10.2024.
